# Process performance and methane production optimizing of anaerobic co-digestion of swine manure and corn straw

**DOI:** 10.1038/s41598-017-09977-6

**Published:** 2017-08-24

**Authors:** Chunlan Mao, Tong Zhang, Xiaojiao Wang, Yongzhong Feng, Guangxin Ren, Gaihe Yang

**Affiliations:** 1 College of Forestry, Northwest A&F University, Yangling, 712100 Shaanxi China; 2 College of Agronomy, Northwest A&F University, Yangling, 712100 Shaanxi China; 3 The Research Center of Recycle Agricultural Engineering and Technology of Shaanxi Province, Yangling, 712100 Shaanxi China

## Abstract

During anaerobic digestion (AD) process, process parameters e.g., VFA, pH, COD removal … and kinetic parameters e.g., hydrolysis rate, lag phase and methane production potential… are the important indicator for illustrating AD process performance, however, the AD process performance based on these parameters remains poorly understood. To estimate process performance focusing on initial pH and substrate composition, the effects of initial pH and swine manure to corn straw ratio on biogas production and these parameters and linkages of these parameters were analyzed. Also, the methane production was optimized. The results revealed that the maximum methane yield and methane production rate were obtained with initial pH 7.5 and SM/CS ratio of 70:30. Kinetic parameters are coupled with process parameters, especially for COD removal rate, VS degradation rate, VFA and pH. Hydrolysis constant positively correlated with pH, COD removal rate and VS degradation rate, then impacted methane production and lag phase. Meanwhile, lag phase and the maximum methane production rate were directly determined by VFA and COD removal rate. The optimum initial pH and SM/CS ratio were 7.15 and 0.62, respectively, with a predicted maximum methane content of 55.12%. Thinking these findings together, they provide a scientific theory for estimating AD performance.

## Introduction

Anaerobic co-digestion has been regarded as one of the more promising options for increasing biogas production because of its better nutrient balance and improved efficiency. It has been extensively applied as an effective waste management and energy production treatment^1, 2^. As reported, due to the high N content in livestock manure and high C content in crop straw, the co-digestion of these organic wastes provides a more suitable substrate-C/N ratio than anaerobic digestion (AD) of either substance alone. The combination is helpful for enhancing AD buffering capacity and for stabilizing the digestion system, and thus increases the process stability and biogas production.

It is well known that AD processes are sensitive to environmental conditions and are easily influenced by operational parameters. To improve the AD efficiency, the influence of temperature, pH, C/N ratio, mixing ratios, additives and other parameters on AD has been studied intensively^3–5^. During the AD process, alkalinity is a better indicator of process performance and directly shows the system’s buffering capacity. This can be managed by adjusting pH value; therefore, pH adjustment could provide a way to improve the self-buffering capacity of AD systems to meet the requirements of the microbial populations^6^. It affects the activities of the specific acidogenic microbial populations and methanogenic bacteria^7^, and consequently influences the process stability^4^. Previous studies have targeted the performance of AD systems operating with different initial pH over a wide range and the optimum initial pH was different. They also included different substrates and operational conditions^8, 9^. Therefore, studies on the effect of these operational parameters have been focused on the analysis of the variation of the process parameters. However, the different operational conditions could result in differences in the substrate characteristics, which would affect AD performance. Moreover, the relevant deep discussions are poor. Many kinetic models have been used to describe mechanisms of the AD-process based on various operational conditions^10–12^. Such work has illustrated that these kinetic parameters are helpful for predicting the time required for acclimatization of bacteria to the new environment, the duration of AD, and the biodegradability of the feed materials. Therefore, the kinetic parameters could be used as indicators to represent the AD performance. However, operational conditions could influence process parameters (e.g., volatile fatty acids (VFA), total ammonia nitrogen (TAN), alkalinity, pH, and organic degradability), and further impact kinetic parameters (e.g., methane production potential, the maximum methane production rate, lag phase, hydrolysis constant). Much less attention has been given to investigation of the AD performance by analyzing how substrate characteristics and environmental conditions are related to process and kinetic parameters, to understand well the effects of operational conditions on methane production.

Hence, in this study, we hypothesized that process parameters are closely correlated to operational conditions such as substrate composition and initial pH. In addition, we also predicted that there is a close relationship between kinetic parameters and process parameters. Therefore, in this work, we focused on (1) the effects of substrate characteristics and initial pH on biogas production, biogas production rate, kinetic parameters, and process parameters; and (2) the linkage of kinetic parameters and process parameters, using swine manure and corn straw as substrates. In addition, the methane content conditions were also optimized.

## Results and Discussion

### Accumulative methane production

**Figure 1:**
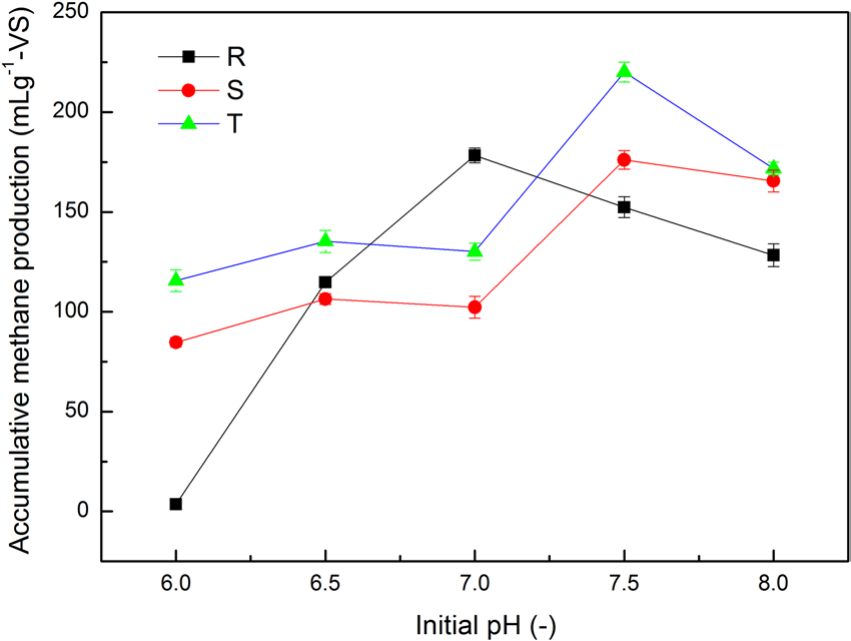
Accumulative methane production of anaerobic co-digestion for each sample. SM: swine manure; CS: corn straw; R: SM/CS ratio of 30:70; S: SM/CS ratio of 50:50; T: SM/CS ratio of 70:30; Error bars are the standard errors in our study.

The effects of the swine manure to crop straw (SM/CS) ratio and initial pH on the total methane production are illustrated in Fig. 1. Three obvious results were found. (1) The accumulative methane production increased with increase in the SM/CS ratio. (2) The accumulative methane production first increased, then decreased with the increase in initial pH. (3) The accumulative methane production was significantly different in each treatment (P < 0.05). The maximum methane yield was achieved in T-group with an initial pH of 7.5. For each SM/CS ratio, the maximum methane yield was obtained at the initial pH of 7.5. The result obtained for T-group (220 mL g^−1^ of volatile solids: VS) was significantly higher (by 30.1 and 20.0%) than for R and T-group, respectively (P < 0.05). The minimum methane yields (4, 85, and 116 mL g^−1^ VS) were obtained at the initial pH of 6.0 for R, S, and T-group, respectively. These results also demonstrated that the SM/CS ratio and initial pH of 7.5 could enhance the organic-to-methane conversion efficiency and the waste utilization.

### Daily specific methane production

**Figure 2:**
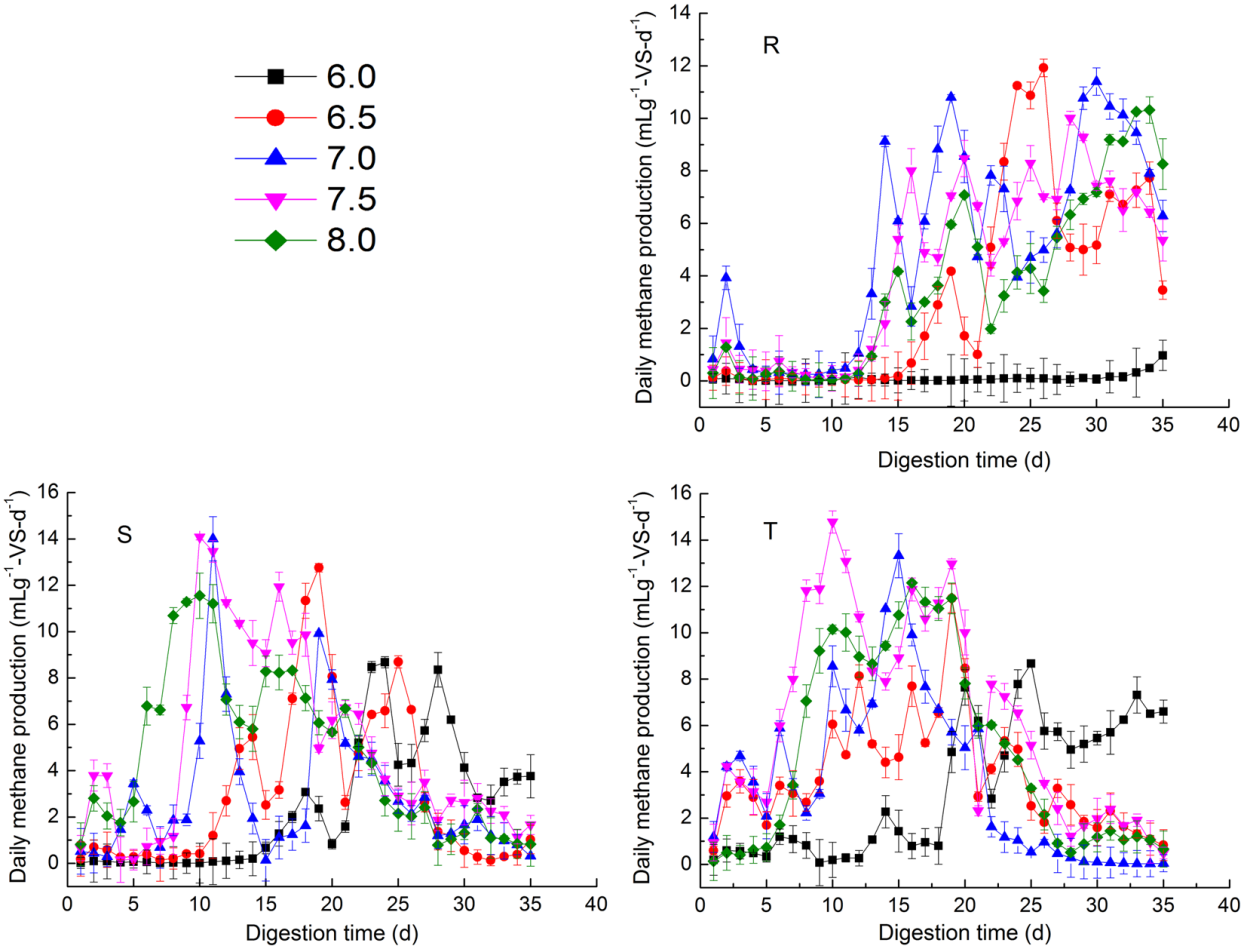
Daily methane production of anaerobic co-digestion for each sample. SM: swine manure; CS: corn straw; R: SM/CS ratio of 30:70; S: SM/CS ratio of 50:50; T: SM/CS ratio of 70:30; Error bars are the standard errors.

**Figure 3:**
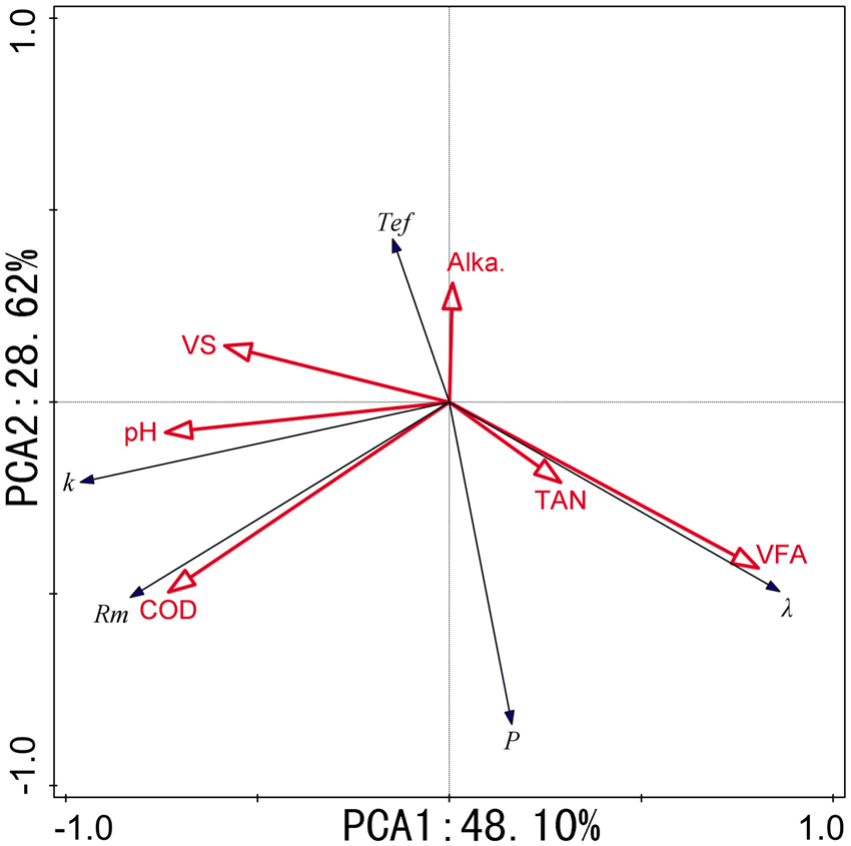
Ordination plots of the results from the principal component analysis (PCA) to identify the relationships of kinetic parameters (Black arrows) and process parameters (Red arrows). TAN: total ammonia nitrogen (mgL^−1^) VFA: volatile fatty acids (mgL^−1^); Alka.: total alkalinity calculated as mg CaCO_3_L^−1^ (mgL^−1^); VS: VS degradation (%); COD: COD removal (%); P: biogas production potential (mLg^−1^-VS); R_m_: the maximum biogas production rate (mLg^−1^-VS-d^−1^); λ: the lag phase (days); T_ef_: the effective biogas production duration calculated by the time period for 90% of total biogas production (T_90_) subtracting λ. k: the observed hydrolysis constant (d^−1^).

The methane production rate reflects the biodegradability and amount of degradable matter. The daily methane production characteristics are shown in Fig. 2. In group R, an obviously prolonged lag phase was found, compared with the other two groups. Rapid methane production of (0.09, 0.38, 3.93, 1.45, and 1.28) mL g^−1^-VS-d^−1^ occurred on the second day, with initial pH of 6.0, 6.5, 7.0, 7.5, and 8.0, respectively. From Day 5 to Day 35, the maximum methane production rate of 11.92 mL g^−1^-VS-d^−1^ for initial pH of 6.5 was observed, which was much higher (by 92, 4, 16, and 13%) than for an initial pH of 6.0, 7.0, 7.5, and 8.0, respectively. For initial pH of 6.0, the maximum methane production rate reached through Day 35 was the lowest value (0.92 mLg^−1^-VS-d^−1^) of all the others, which could indicate failure of the AD system. Furthermore, although the maximum methane yield occurred with an initial pH of 7.5, the associated maximum methane production rate was lower than with initial pH of 6.5. This implied that low initial pH could improve the biodegradation, hydrolysis, and acidification rates (which might cause VFA accumulation), then inhibit methanogens activity. As shown in R-group (Fig. 3), at initial pH 6.0, the daily methane production showed no obvious peak during anaerobic digestion until Day 35. While when the initial pH was higher, the daily methane production peak was obtained earlier. Hence, when initial pH was adjusted to 6.0, a longer lag-phase was found. As the AD process progressed, methanogen activity recovered, the accumulated VFA was sufficient for methane production, and the maximum methane production rate was observed. However, the AD had to be prolonged to improve methane production, and the input cost would be increased. An initial pH of 7.5 was more helpful for relieving hydrolytic inhibition of the digestion system and more suitable for microbial metabolism; therefore, the system start-up and methane production were accelerated. Daily methane production at an initial pH of 6.0 did not exhibit significant fluctuations and maintained a low level after three days of incubation. Hence, the anaerobic reactors with initial pH of 6.0 failed. The lag phase was day 15, 12, 13, and 13 for initial pH of 6.5, 70, 7.5, and 8.0, respectively.

In the reactors of S-group, the highest methane production rate (14.08 mLg^−1^-VS-d^−1^) was obtained at the initial pH of 7.5. This result was similar to that with initial pH 7.0 (14.01 mLg^−1^-VS-d^−1^), and higher (by 38, 9, and 18%) than that for initial pH of 6.0, 6.5, and 8.0, respectively. The lag phase was (14, 10, 3, 3, and 4) d for initial pH 6.0–8.0 taking 5 as the gradient, respectively. A similar trend was found in T-group, of which the maximum methane production rate (14.78 mLg^−1^-VS-d^−1^) was observed at initial pH of 7.5 on Day 10. It was significantly higher (by 41, 22, 10, and 18%) than that for initial pH 6.0, 6.5, 7.0, and 8.0, respectively. The lag phase for each initial pH value was five days.

The present investigation indicated that (1) high methane production can be achieved with substrate composition of 70:30 SM/CS. (2) When the ratio was set at 50:50 or 70:30 the methane production at initial pH of 7.0 was near the results obtained with initial pH of 7.5. The results in this work were similar to those in previous studies^4^. However, when the initial pH was higher, methane production decreased. Based on the aforementioned information, the differences between results at the five initial pH levels were significant. (3) The initial pH of 6.0 showed the lowest efficiency. There are two possible explanations for this. One might be that more H_2_ was produced when the initial pH was 6.0. As a byproduct during acidification of the organics, H_2_ is a thermodynamically unfavorable intermediate during anaerobic methanogenesis because it may impede the decomposition of organic acids^13^, thereby causing acid inhibition. Therefore, less methane was produced and start-up was delayed. In addition, according to the previous study, when the initial pH was 6.0, the hydrolytic enzyme activities might be highest, resulting in the highest VFA concentration^8^, and causing the growth rate of methanogens to be greatly reduced^8^. Initial pH values of 7.0 and 7.5 were ideal for methane production, and allowed higher and steadier methane production to be achieved. This was consistent with other studies, which found that methane concentration and biomethanization increased when the initial pH was 7.0–7.6^14^. Moreover, the high C:N ratio of the CS could be the best explanation for the low methane yield in R-group. This is because extremely high or low C:N ratios would inhibit methane production^15^, which would favor accumulation of VFA and ammonia, and result in toxic inhibition of methanogenic archaea^16^.

### Effect of the SM/CS ratio and initial pH on process performance

It was postulated that the process performance observed in the present study was significantly influenced by the SM/CS ratio and initial pH. If this is the case, then the lack of trace elements and the differences in the high C/N ratio and VS caused by the SM/CS ratio and the initial pH, could be deemed the main factors affecting process performance. In the present study, this postulation was tested and proved. According to the above information, we chose the initial pH of 7.5 and T-group to analyze the effects of the SM/CS ratio and initial pH on AD performance, respectively.

The variation in these process parameters (e.g., COD removal rate, VS degradation rate, TAN, VFA, alkalinity, and pH) is presented in Table 1. Ignoring the effect of initial pH; the COD removal rate, VS degradation rate, final TAN concentration, and pH increased with increased SM/CS ratio, while the final VFA concentration and alkalinity decreased. It was revealed that 70% SM content enhanced biodegradability and improved the system buffer capacity. Furthermore, no significant difference was found in the COD removal rates of the three groups. The lowest VS degradation rate (7.5%), final TAN concentration (365.1 mgL^−1^), and final pH (5.4) and highest final VFA concentration (418 mgL^−1^) in R-group showed low biodegradation, an unbalanced C:N ratio, and low buffer capacity. These differences among the three groups were mainly caused by (1) the lack of trace nutrients in the reactors due to lower SM concentration, which resulted in reduction of methanogenic activity; (2) high carbohydrates levels in R-group, leading to imbalance of C/N and conversion of carbon to excessive acids that caused acid inhibition before methanogenesis; (3) more phytic acid in the CS, resulting in the accumulation of propionic acid, and causing process failure at a concentration of >1 g/L^17^; and (4) concentrations of N and easily biodegradable constituents in the feedstock increased as the SM/CS ratio increased from 30:70 to 70:30, which probably enabled greater stability in the process. This suggested that providing a quantity of SM appropriate to avoid acidification of CS was important for obtaining better AD performance.

**Table 1:** Effects of SM/CS ratio and initial pH on process parameters of anaerobic co-digestion of SM and CS.

Item	COD removal (%)	VS degradation (%)	Final VFA	Final TAN	Final alkalinity	Final pH
**Effect of SM/CS ratio at initial pH 7.5**						
R	90.3(1.6)a	7.5(1.6)b	418.0(52.4)a	365.1 (32.3)b	1260(28.7)a	7.4(0.1)b
S	87.6(1.7)a	16.7(2.2)a	261.0(53.4)b	557.8(34.5)a	1160(35.9)b	7.5(0.1)b
T	92.2(1.5)a	23.8(1.9)a	241.6(52.3)b	677.6(34.2)a	740(30.4)c	7.6(0.2)a
**Effect of initial pH at SM/CS ratio 70:30**						
6.0	70.1(1.4)c	11.5(0.4)d	585.5(19.1)a	711.5(70.5)a	840(57.1)bc	7.3(0.1)b
6.5	64 (1.4)d	15.5(0.2)c	255.7(50.5)b	679.8(56.5)ab	900(37.6)ab	7.5(0.1)ab
7.0	87.8 (1.5)bc	17(0.1)b	204.6(21.9)b	557.8(68)ab	940(14.2)ab	7.4(0.1)ab
7.5	89.6 (1.5)ab	20.4 (0.2)a	241.6(14.5)b	677.6(76.5)bc	740(22)c	7.6(0.1)a
8.0	92.1(1.6)a	10.1 (0.5)e	209.9(19.4)b	577.92(20.1)c	960(22.2)a	7.5(0.1)ab

SM: swine manure; CS: corn straw; R: SM/CS ratio of 30:70; S: SM/CS ratio of 50:50; T: SM/CS ratio of 70:30; the letters indicate significant difference at a level of 0.05. The values in parenthesis are the standard errors.

Considering the effect of initial pH only, it was found that organic degradation increased with higher initial pH, as indicated by a significantly higher COD removal rate and VS degradation rate. Therefore, the COD removal rate and VS degradation rate, and the final VFA and TAN concentrations achieved at initial pH 7.5, were higher than those obtained at initial pH of 6.5, 7.0, and 8.0. That the highest methane yield was achieved at initial pH of 7.5 verified these results. Compared with final pH, it shown that the process in all trials could recover to the normal condition (pH > 6.5). Reactors with initial pH of 6.0 showed significant differences and opposite trends in the process parameters than did reactors at other pH values. Therefore, controlling the initial pH could be a useful method for achieving the optimal alkaline condition needed to meet the requirements of the microbial populations and to improve biodegradation in the AD system. It is well known that in the first few days, the self-buffering systems of AD (such as CO_3_^2−^/HCO_3_^−^ and NH_4_^+^/NH_3_·H_2_O) have not yet formed, resulting in decreased pH due to the generation of VFAs. The buffer system recovers and pH increases as a result of the consumption of VFAs and production of biogas. The pH in all reactors ranged from 4.8 to 7.6 (6.5–7.4 during stable operation). The pH values could serve to assay hydrolysis during the anaerobic process^18^. Therefore, the initial pH of 6.0 would be more suitable for organic degradation (hydrolysis rate) and rapid growth of acidogenic bacteria, but would then impede the adaptation and recovery of activity of methanogenic bacteria. From this situation, hydrolysates accumulated that could inhibit the AD start-up. It is well known that if any one of stages is inhibited then the overall processes becomes less efficient and effective, and might even collapse because of accumulated intermediate products^19^. This is especially true in the batch digesters from which products were not removed during the process. The pH value of the reactor with initial pH of 6.0 did not recover to the normal pH (6.5) until Day 25, while others returned to the normal pH in a short time. These results indicated that at the SW/CS ratio of 70:30, and the initial pH levels of 6.5–7.5, although total VFAs accumulated and the pH dropped during the initial phase of AD, the accumulated VFAs were consumed and the acidification was thereby reversed (pH rose again).

It is well known that CS and SM are both characterized by high carbon and nitrogen content. Therefore, the main differences among R, S, and T-group were the differences in the C:N ratio. In the present study, the C:N ratio was 43.5:1, 35.4:1, and 27.1:1 for R, S, and T group, respectively, and the group differences were significant (P < 0.01). However, the optimal C:N ratio for the anaerobic digestion process is 20:1 to 30:1. Extremely high C:N ratios could result in AD process inhibition. This is the reason that R-group obtained the lowest biogas production. Therefore, in order to explain the effects of the C:N ratio and initial pH, and to determine the correlation between these factors and process parameters, the correlations of C:N and initial pH with the process parameters were analyzed. As shown in Table 2, there is a significant negative correlation between the C:N ratio and TAN (P < 0.01) and a significant positive correlation between the C:N ratio and VFA (P < 0.01). Thus, the high C:N ratio in R-group led to low TAN and VFA accumulation. As a result, the pH decreased (which showed a significant negative correlation with the C:N ratio, P < 0.05), anaerobic digestion was inhibited, lag time was prolonged, and biogas production was also decreased (see Figs 1 and 2). However, in order to improve the stability of the system, the self-buffering capacity was increased to improve the system alkalinity. Hence, a significant positive correlation between the C:N ratio and Alk (P < 0.01) was found. Furthermore, a high C:N ratio indicated high CS content in this study. High cellulose, hemicellulose, and lignin content resist biodegradation; hence, low VS degradability was obtained, which negatively correlated with the C:N ratio (P < 0.01). Therefore, for T-group, better anaerobic digestion performance was achieved. Moreover, for the initial pH, the results also revealed that as initial pH increased, COD removal increased and the positive correlation was significant (P < 0.01). This is because when the initial pH increased (from 6.0 to 7.5) the biodegradation of organics increased; thus, the demand for oxygen to oxidize the organics increased and high COD resulted. In addition, the VFA showed a negative and significant correlation (P < 0.01) with initial pH. This is why at the initial pH of 6.0, a high concentration of VFA, low pH, and minimum biogas production were found.

**Table 2:** Pearson’s correlation coefficients of C:N ratio and initial pH with process parameters.

Item	TAN	VFA	Alka.	pH	COD removal	VS degradation
C:N ratio	−0.897^**^	0.737^*^	0.943^**^	−0.796^*^	0.137	−0.875^**^
Initial pH	−0.480	−723^**^	0.122	0.505	0.849^**^	0.085

Statistically significant values are indicated by symbols: **P < 0.01; *P < 0.05.

TAN: total ammonia nitrogen (mgL^−1^) VFA: volatile fatty acids (mgL^−1^); Alka.: total alkalinity calculated as mg CaCO_3_L^−1^ (mgL^−1^).

### Kinetics study

For this section, according to the methane yield, we analyzed the model results of T-group with initial pH in the range 6.0–8.0.

### Lag phase, maximum methane production rate, and methane production potential

The modified Gompertz model (see Eq. 1)^20^ was used to fit the accumulative methane production data obtained from the AD experiments to predict the methane potential.

1 $$M({\rm{t}})=P\cdot \exp \{-\exp [\frac{{R}_{m}\cdot e}{P}\cdot (\lambda -t)+1]\}$$

where *M* is the accumulative methane production (mL g^−1^-VS) at AD time *t* (d), *P* is the final accumulative methane production (mL g^−1^ -VS), *R*_*m*_ is the maximum methane production rate (mLg^−1^-VS-d^−1^), *e* is Euler’s constant (2.7183), and *λ* is the lag phase time (days).

The estimated kinetic parameters are shown in Table 3. These indicated that the modified equation can be used to present methane production and lag-phase with correlation coefficients from 0.996 to 0.999. The maximum methane production potential (*P*) and the maximum methane production rate (*R*_*m*_) in all trials were first increased; then decreased with increase in the initial pH, while the lag phase (λ) showed the opposite tendency. The maximum *P* of 230.5 mL g^−1^ VS was obtained at initial pH 6.0, which similar was to that with initial pH 7.5, but significantly higher those obtained at other initial pH. However, *R*_*m*_ was the lowest and λ was the longest with initial pH 6.0; thus, prolonged AD was needed to achieve the predicted potential methane production. This was unavailable in the practical application. Initial pH 7.5 resulted in the maximum methane production potential (except for initial pH of 6.0), and the maximum methane production rate and shortest λ. Furthermore, the duration of the lag phase demonstrates the buffer capacity of the fermentation liquor and the time needed for bacterial acclimatization to the changed environment. Therefore, optimizing the initial pH could improve the buffer capacity of the AD system and microbial activity, and subsequently increase methane production.

**Table 3:** Results obtained from first-order model and modified Gompertz model of T group with a SM/CS ratio of 70:30.

Parameters	6.0	6.5	7.0	7.5	8.0
**Modified Gompertz model**					
P	230.5(5.7)a	149.8(4.3)c	137(5.0)c	230(5.4)a	176(5.1)b
R_m_	6.4(0.2)c	6.5(0.4)c	8.8(0.3)b	12.12(0.3)a	11.9(0.5)a
λ	16.9(0.1)a	5.4(0.1)c	5.2(0.2)cd	4.8(0.2)d	7.7(0.2)b
T_ef_	16.1(0.3)c	17.6(0.3)b	14.8(0.2)d	19.2(0.5)a	15.3(0.1)cd
R^2^	0.996	0.997	0.988	0.999	0.999
**First-order model**					
k	0.0717(0.008)d	0.1156(0.014)c	0.1595(0.01)b	0.2121(0.011)a	0.1596(0.012)b
R^2^	0.637	0.841	0.931	0.880	0.929

SM: swine manure; CS: corn straw; P: the maximum accumulative methane production predicted using modified Gompertz model (mLg^−1^-VS); R_m_: the maximum methane production rate (mLg^−1^-VS-d^−1^); λ: the lag phase (days); T_ef_: the effective biogas production duration calculated by the time period for 90% of total biogas production (T_90_) subtracting λ. k: the observed hydrolysis constant (d^−1^); The letters indicate significant difference at a level of 0.05. The values in parenthesis are the standard errors.

The effective methane production time (*T*_*ef*_) was calculated by subtracting *λ* from the time required to achieve 90% methane yield (*T*_90_). This could be an indicator to predict the anaerobic digestion period, conversion efficiency of organics to biogas, and the methane production rate based on *λ* and methane yield. Compared with each initial pH, it was found that initial pH 7.5 showed the maximum *T*_*ef*_ of 19.2 d, but the lag phase was the shortest and methane yield was the highest. This implied that this was the highest organic-to-methane conversion efficiency, and could be proved by the maximum methane production rate. Longer *T*_*ef*_ with longer *λ* might indicate a shorter anaerobic digestion period and low degradability, methane production rate, and longer duration of AD. This was found for initial pH 6.0 and 6.5, which had the longest *T*_*ef*_ (16.1 and 17.6 d, respectively).

### Hydrolysis constant

The hydrolysis constant (*k*) obtained from the first-order kinetic model was used to evaluate the substrate suitability and estimate the process rate-limiting stage. As shown in Table 3, the AD data for five initial pH values were well fitted by a first-order kinetic model (*R* ^2^ ranged from 0.637–0.929). Compared with the fitted results, the values of k showed a trend similar to those of P and R_m_. Initial pH 7.5 resulted a maximum k of 0.21 d^−1^, which was significantly higher than those obtained from other initial pH, while the minimum k (0.07 d^−1^) was found at initial pH 6.0. In this case, *k* describes the velocities of degradation and methane production^21^; therefore, high *k* represents high rates of degradation and methane production. In this study, our results provide evidence that adjusting the initial pH to 7.5 improves biodegradation and methane production.

### Effects of process parameters on the kinetic parameters

According to the above results, we concluded that these kinetic parameters could be influenced by the process parameters. Therefore, the effects of these process parameters (COD removal rate, VS degradation rate, pH, VFA, TAN, and alkalinity) on kinetic parameters (*P*, *R*_*m*_, *λ*, *k* and *T*_*ef*_) were examined based on principal component analysis and Pearson correlation analysis (see Fig. 3 and Table 4). It was found that that VFA, COD removal rate, VS degradation rate, and pH were the main factors that influenced the kinetic parameters (together explaining 98.6% of the total variation) (Fig. 3). In this case, *k* was significantly increased by pH (r = 0.776, P < 0.01), the COD removal rate (r = 0.796, P < 0.01), and the VS degradation rate (r = 0.636, P < 0.01), but decreased by VFA (r = −0.625, P < 0.05). *R*_*m*_ was positively correlated with pH (r = 0.624, P < 0.05), and the COD removal rate (r = 0.908, P < 0.01); λ was positively correlated with VFA (r = 0.928, P < 0.01) and negatively correlated with the VS degradation rate (r = −0.626, P < 0.05). Furthermore, *k* could significantly improve R_m_ (r = 0.872, P < 0.01) and P (r = 0.872, P < 0.01), and decrease λ (r = −0.721, P < 0.01). Comparing these correlations, it was concluded that (1) high substrate biodegradation and buffer capacity improved the hydrolysis constant, and thus improved the methane production rate and methane yield, while decreasing the lag phase; (2) λ was closely correlated with VFA, because a high VFA concentration inhibited microbial activity. This prolonged the time for bacterial acclimatization to the changed environment so the lag phase was prolonged. However, the maximum VFA was obtained at the SM/CS ratio of 30:70 and initial pH 6.0, while the SM/CS ratio of 70:30 and initial pH 7.5 achieved the maximum COD removal rate and VS degradation rate. Therefore, use of the SM/CS ratio of 70:30 and initial pH 7.5 could increase the hydrolysis constant and methane production, as well as shorten the lag phase and AD duration.

**Table 4:** Pearson’s correlation coefficients of kinetic parameters with process parameters.

	TAN	VFA	Alka.	pH	COD removal	VS degradation	P	R_m_	λ	T_ef_
VFA	0.629^*^									
Alka.	−0.204	−0.128								
pH	0.321	−0.347	0.123							
COD removal (%)	−0.426	−0.477	0.114	0.406						
VS degradation (%)	0.116	−0.367	−0.454	0.463	0.185					
P	0.602^*^	0.618^*^	−0.568^*^	0.130	0.061	0.043				
R_m_	−0.245	−0.509	−0.053	0.624^*^	0.908^**^	0.239	0.220			
λ	0.387	0.928^**^	−0.053	−0.539	−0.380	−0.626^*^	0.542^*^	−0.455		
T_ef_	0.568^*^	−0.076	−0.278	0.429	−0.510	0.450	0.064	−0.181	−0.330	
k	−0.150	−0.625^*^	−0.077	0.776^**^	0.796^**^	0.636^**^	0.872^**^	0.872^**^	−0.721^**^	0.088

Statistically significant values are indicated by symbols: **P < 0.01; *P < 0.05.

TAN: total ammonia nitrogen (mgL^−1^) VFA: volatile fatty acids (mgL^−1^); Alka.: total alkalinity calculated as mg CaCO_3_L^−1^ (mgL^−1^); P: biogas production potential (mLg^−1^-VS); R_m_: the maximum biogas production rate (mLg^−1^-VS-d^−1^); λ: the lag phase (days); T_ef_: the effective biogas production duration calculated by the time period for 90% of total biogas production (T_90_) subtracting λ. k: the observed hydrolysis constant (d^−1^).

### Model of methane content

The methane content during the AD process not only shows biogas productivity, but also reflects digestion performance. For these reasons, it is essential to evaluate the relationship between the methane content and operational parameters (initial pH level and substrate combination ratio). The methane content data were shown in Supplementary Information (Tables S1, S2 and S3). The results from significance analysis of coded factors are shown in Table 5. Linear, quadratic, and cross product analyses all indicated significant differences. This also indicates that initial pH and the substrate combination-ratio affect the biogas production and methane content significantly. The methane content model was fitted in terms of the coded factors in Eq. (2):

2 $$Y={52.67}+{7.73}\,{X}_{1}+{6.68}\,{X}_{2}-{6.8}\,{X}_{1}{X}_{2}-{12.54}\,{X}_{1}^{2}-{4.88}\,{X}_{2}^{2}$$

where *Y* is methane content, *X*_*1*_ is the initial pH value, and *X*_*2*_ is SM/CS ratio. The *R* ^2^ obtained using Eq. (4) was 0.91, which indicates that most of the data obtained could be explained by the model, and that an ideal fit existed between the experimental and predicted values. Methane content can be obtained according to the quadratic response surface of initial pH and SM/CS ratio. The predicted maximum value of the methane content was 55.12% at initial pH of 7.15 and SM/CS ratio of 0.62, which were the optimum conditions determined for anaerobic co-digestion of SM and CS. A test experiment was performed subsequently and the maximum methane content was 56.38%, which was in accordance with the predicted value.

**Table 5:** ANOVA for Response Surface Quadratic Model of methane content.

Source	Sum of Squares	df	Mean Square	F value	P value	Significance
Model	1617.54	5	323.51	14.10	0.0007	**
*X*_1_	447.76	1	447.76	8.20	0.0057	**
*X*_2_	446.22	1	446.22	9.45	0.0096	**
*X*_1_ *X*_2_	231.2	1	231.2	10.81	0.0004	**
*X*_12_	412.97	1	412.97	12.28	0.0006	**
*X*_22_	79.38	1	79.38	8.88	0.0023	**

where *X*_1_ is the initial pH value, *X*_2_ is the ratio of SM/CS, **meant extremely significant level.

## Conclusions

SM/CS ratio and initial pH significantly affected the AD process performance. Kinetic parameters are coupled with process parameters, especially for COD removal rate, VS degradation rate, VFA and pH. Hydrolysis constant positively correlated with pH, COD removal rate and VS degradation rate, then impacted methane production and lag phase. Meanwhile, lag phase and the maximum methane production rate were directly determined by VFA and COD removal rate. The optimum initial pH and SM/CS ratio were 7.15 and 0.62, respectively, with a predicted maximum methane content of 55.12%. Thinking these findings together, they provide a scientific theory for estimating anaerobic digestion performance.

## Methods

### Collection of feedstock and inoculum

SM and air-dried CS were obtained from a local village in Yangling, China. CS was ground into 2–3 mm pieces prior to batch experiments. Anaerobic inoculum was collected from a mesophilic anaerobic reactor in the same village. All samples were individually homogenized and stored. The physicochemical properties of the substrates and inoculum used in this study were determined according to the previous study^4^. Due to the high content of nitrogen, SM had a lower C/N ratio (17.85 ± 0.21) than CS (82.26 ± 1.51). Comparing with SM and inoculum, SM had a lower pH value (6.4 ± 0.12) than inoculum (8.4 ± 0.18).

### Experimental design

All the experiments were conducted in self-designed batch digesters fabricated from 1 L glass conical bottles^22^. Each AD reactor contained 200 mL inoculum and 500 mL substrate and was tightly closed with a rubber septum and screw cap. Three substrate combination ratios (SM/CS) of 30:70 (R group), 50:50 (S group) and 70:30 (T group) (% of total feedstock measured as wet weight (w/w)) were tested under mesophilic (35 ± 1 °C) condition for 35 days with the initial total solid (TS) concentration of 8%^23^. Initial pH values of each group were adjusted to 6.0, 6.5, 7.0, 7.5, and 8.0 using 5 mol/L NaOH and 5 mol/L HCL. Each sample was mixed for 3–5 min before AD to obtain a homogeneous mixture and was flushed with nitrogen gas for about 2 min to assure AD conditions before the batch experiments. All reactors were shaken manually for 1 min each day during AD process. All samples were analyzed in triplicate.

### Analytical techniques

The total solid (TS), volatile solid (VS), total ammonium nitrogen (TAN), total organic carbon (TOC), total organic nitrogen (TON), alkalinity, total chemical oxygen demand (TCOD) and total volatile fatty acids (VFAs) were determined in accordance with APHA Standard Methods (1995). The methane volume was measured by using the water displacement method each day throughout the AD process. The pH and methane content were measured using a fast biogas analyzer (Gas-board-3200P).

### Kinetic study

#### Modified Gompertz model

A modified Gompertz model showed as Eq. (1) to determine the methane production potential, maximum methane production rate and the duration of the lag phase of the reaction with available experimental results^20^.

#### First-order model

The first-order kinetic model (Eq. (3)) reported by ref. 22 was used to description the hydrolysis constant.

3 $${M}_{(t)}={M}_{max}\cdot [1-exp(-kt)]$$

where *M*_*(t)*_ is the accumulative methane production (mLg^−1^-VS) at an anaerobic digestion time t (d), *M*_*max*_ is the maximum accumulative methane production (mLg^−1^-VS), and *k* is the hydrolysis constant (d^−1^).

### Statistical analyses

Principal component analysis (PCA) was used to identify the main operational conditions affected kinetic parameters. The PCA was performed using the CANOCO 4.5 software package. A forward selection procedure was implemented to select a sub-set of operational conditions. Pearson correlation analysis was performed to determine the relationship between kinetic parameters and operational conditions (SPSS.20). Additionally, to test for differences in the methane production and those parameters of each sample in relation to operational conditions, we used one-way ANOVA, analysis of variance of means, and Duncan’s Multiple Range Test (DMRT).

## Electronic supplementary material

Supplementary Information
