# Supplementary material for: Process performance and methane production optimizing of anaerobic co-digestion of swine manure and corn straw
Source: Sci Rep. 2017 Aug 24;7:9379. doi: 10.1038/s41598-017-09977-6 (PMC5571176; doi:10.1038/s41598-017-09977-6)
Supplement: Supplementary file 1 — Supplementary Information [file 41598_2017_9977_MOESM1_ESM.doc]

**Supplementary Information**

Process performance and methane production optimizing of anaerobic co-digestion of swine manure and corn straw

Chunlan Mao^a,c^, Tong Zhang^b,c^, Xiaojiao Wang^b,c^,,Yongzhong Feng^b,c,*^, Guangxin Ren^b,c^, Gaihe Yang^b,c^

a, College of Forestry, Northwest A&F University, Yangling, 712100 Shaanxi, China

b, College of Agronomy, Northwest A&F University, Yangling, 712100 Shaanxi, China

c, The Research Center of Recycle Agricultural Engineering and Technology of Shaanxi Province, Yangling, 712100, Shaanxi, China

**Corresponding Author:** Yongzhong Feng

Tel: (86) 029-87092265; Fax: (86) 029-8709 2265;

E-mail address: fengyz@nwsuaf.edu.cn.

Table S1 Methane content in biogas (%) of R-group: SM/CS ratio of 30:70

| Initial pH | 6.0 | 6.5 | 7.0 | 7.5 | 8.0 |
| --- | --- | --- | --- | --- | --- |
| 1 | 0.9 | 1.5 | 9.3 | 4.6 | 3.8 |
| 2 | 1.1 | 1.8 | 18.5 | 6.2 | 6.3 |
| 3 | 1.2 | 2.6 | 21.2 | 6.8 | 12.4 |
| 4 | 1 | 4.8 | 20.4 | 12.8 | 10.3 |
| 5 | 2 | 5.8 | 23.8 | 14.3 | 13.6 |
| 6 | 2.3 | 5.8 | 24.6 | 16.2 | 13.8 |
| 7 | 2.6 | 6.1 | 32.1 | 18.7 | 14.9 |
| 8 | 2.9 | 6.5 | 39 | 28 | 14.6 |
| 9 | 3.2 | 7.2 | 39.4 | 28.1 | 18 |
| 10 | 3.6 | 7.4 | 41.5 | 30.1 | 18.3 |
| 11 | 3.6 | 7.4 | 45.2 | 33.6 | 20.1 |
| 12 | 3.5 | 8.5 | 49.1 | 38.1 | 31.4 |
| 13 | 3.2 | 8.3 | 56.3 | 41 | 42.4 |
| 14 | 3.2 | 9.1 | 58.3 | 45 | 51 |
| 15 | 2.4 | 9 | 58 | 45.9 | 51.2 |
| 16 | 2.4 | 18.2 | 60.2 | 46.1 | 52.5 |
| 17 | 2.7 | 29.1 | 61.6 | 46.9 | 55.1 |
| 18 | 3.6 | 34.2 | 62.4 | 49.5 | 58.4 |
| 19 | 4.7 | 52.5 | 64.6 | 50.2 | 62 |
| 20 | 5.3 | 61.4 | 63.5 | 50 | 62.3 |
| 21 | 5.3 | 61 | 63 | 53.6 | 61 |
| 22 | 5.1 | 62.4 | 62.4 | 54.1 | 61.6 |
| 23 | 5 | 66 | 62.6 | 59.4 | 50.4 |
| 24 | 4.9 | 66.4 | 63.6 | 71 | 48.2 |
| 25 | 4.9 | 65 | 62.7 | 71.6 | 47.4 |
| 26 | 5 | 63.2 | 61.2 | 70.4 | 47 |
| 27 | 6.4 | 58.1 | 58.2 | 67.2 | 48 |
| 28 | 6.9 | 52.9 | 57.5 | 63.1 | 48.4 |
| 29 | 7.6 | 43.2 | 54.6 | 58.5 | 49 |
| 30 | 9.7 | 40.9 | 55.4 | 52.5 | 49.3 |
| 31 | 10 | 42.5 | 55.1 | 52 | 50.2 |
| 32 | 11.4 | 46.2 | 52.6 | 54.6 | 52.4 |
| 33 | 18.6 | 49.1 | 50.1 | 57.8 | 55.6 |
| 34 | 25.4 | 53 | 49.2 | 61.5 | 59.4 |
| 35 | 28.9 | 53.8 | 46.7 | 62.5 | 58.9 |

These values are the average value of triplicate.

Table S2 Methane content in biogas (%) of S-group: SM/CS ratio of 50:50

| Initial pH | 6.0 | 6.5 | 7.0 | 7.5 | 8.0 |
| --- | --- | --- | --- | --- | --- |
| 1 | 0.5 | 4.6 | 9.2 | 10.5 | 9.8 |
| 2 | 1.1 | 8.2 | 11.6 | 15.5 | 14.6 |
| 3 | 1.3 | 9.4 | 15.5 | 21.6 | 18.2 |
| 4 | 2.1 | 12.8 | 23.6 | 32.7 | 27.2 |
| 5 | 3.3 | 14.9 | 32.6 | 42.8 | 36.5 |
| 6 | 3.4 | 21.1 | 35.8 | 50.6 | 45.1 |
| 7 | 3.3 | 26.6 | 42.7 | 56.3 | 51.7 |
| 8 | 4.6 | 31.6 | 47.3 | 60.1 | 56 |
| 9 | 5.1 | 33 | 55.7 | 61.5 | 62 |
| 10 | 5.3 | 33.4 | 63.9 | 60.5 | 62.7 |
| 11 | 6.3 | 40.1 | 63 | 58.2 | 61.6 |
| 12 | 9.2 | 46.2 | 60.2 | 55.7 | 57.2 |
| 13 | 11.5 | 50.1 | 56.8 | 54.7 | 51.2 |
| 14 | 14.2 | 52.7 | 53.5 | 54 | 48.3 |
| 15 | 20.7 | 53.4 | 48.6 | 52.8 | 47.9 |
| 16 | 26.4 | 58.2 | 47.7 | 51.6 | 47 |
| 17 | 32.4 | 64.6 | 46 | 50.3 | 48 |
| 18 | 39.7 | 70.1 | 48.3 | 48.8 | 49.6 |
| 19 | 43.8 | 71 | 50.2 | 47.1 | 50 |
| 20 | 52.8 | 71.1 | 55.2 | 45.1 | 50.4 |
| 21 | 54.3 | 71 | 55 | 46 | 50 |
| 22 | 55.2 | 64.6 | 52.6 | 46.3 | 47.4 |
| 23 | 50.1 | 58.3 | 50.2 | 48.1 | 45.8 |
| 24 | 48.3 | 55.3 | 48.1 | 48 | 43.1 |
| 25 | 44.2 | 53 | 45.7 | 48.4 | 42 |
| 26 | 42.1 | 53.7 | 47.2 | 49 | 43.1 |
| 27 | 46.4 | 57.3 | 50.1 | 50 | 46.7 |
| 28 | 50.6 | 61 | 51.3 | 51.7 | 50.1 |
| 29 | 53.6 | 62.6 | 52 | 55 | 52 |
| 30 | 65.6 | 61.3 | 52.6 | 55.7 | 52.8 |
| 31 | 65.3 | 60.4 | 50.5 | 52 | 52 |
| 32 | 65 | 58.2 | 46.1 | 51.5 | 51.2 |
| 33 | 62.5 | 56.7 | 44.6 | 49.1 | 50.1 |
| 34 | 59.4 | 56.4 | 42.1 | 47 | 50 |
| 35 | 57.7 | 50.7 | 41.8 | 47.6 | 50.8 |

These values are the average value of triplicate.

Table S3 Methane content in biogas (%) of T-group: SM/CS ratio of 70:30

| Initial pH | 6.0 | 6.5 | 7.0 | 7.5 | 8.0 |
| --- | --- | --- | --- | --- | --- |
| 1 | 4.2 | 10.6 | 15.7 | 11.8 | 1.3 |
| 2 | 5.8 | 21.6 | 26.7 | 17.4 | 2.1 |
| 3 | 7.3 | 30.5 | 35.8 | 23.8 | 2 |
| 4 | 9.1 | 39 | 43 | 28.1 | 3.5 |
| 5 | 10.5 | 39.9 | 43.5 | 32.6 | 4.8 |
| 6 | 14.5 | 40.1 | 43 | 36.8 | 12.7 |
| 7 | 16.3 | 43.6 | 43.7 | 43.7 | 19.1 |
| 8 | 18.4 | 51.6 | 44.9 | 50.1 | 31.8 |
| 9 | 22.5 | 55.6 | 46.2 | 59 | 43.7 |
| 10 | 27.4 | 59.6 | 47.7 | 59.1 | 56.6 |
| 11 | 27 | 59 | 47 | 59 | 56 |
| 12 | 28.4 | 58.4 | 48.1 | 60.4 | 55.1 |
| 13 | 32.5 | 58 | 50.7 | 60 | 53.2 |
| 14 | 38.5 | 57.8 | 52 | 61.2 | 54.8 |
| 15 | 42 | 57.7 | 56.5 | 62 | 56.4 |
| 16 | 42 | 57.2 | 56 | 60.6 | 56 |
| 17 | 46.5 | 57 | 59.1 | 59.1 | 55.2 |
| 18 | 48.6 | 56.7 | 59 | 58 | 55.1 |
| 19 | 51.4 | 57 | 60.5 | 57.9 | 55 |
| 20 | 56.8 | 57.9 | 61.9 | 56.2 | 53 |
| 21 | 58.4 | 56.4 | 62 | 56.1 | 53.4 |
| 22 | 56 | 56 | 61 | 56.9 | 52.1 |
| 23 | 55.3 | 55.1 | 57.3 | 58 | 50.3 |
| 24 | 55 | 54 | 55 | 59 | 50 |
| 25 | 57.4 | 54 | 55 | 59.5 | 48.8 |
| 26 | 57 | 53.1 | 54.7 | 58.3 | 48 |
| 27 | 55.3 | 52.5 | 52.6 | 57 | 48.1 |
| 28 | 52.6 | 52 | 51 | 57.5 | 49 |
| 29 | 45.4 | 51.5 | 46.8 | 55.9 | 49.4 |
| 30 | 46.2 | 51 | 42.6 | 55.8 | 49.6 |
| 31 | 46 | 44.5 | 42 | 54.2 | 49.1 |
| 32 | 48.1 | 40.1 | 41.8 | 52.7 | 48.2 |
| 33 | 50 | 38.5 | 42.7 | 49.1 | 48 |
| 34 | 50.1 | 37 | 43 | 46 | 47 |
| 35 | 50.4 | 37.1 | 44.1 | 46 | 47.9 |

These values are the average value of triplicate.
